# Intramyocardial dissection of the left ventricle and post-myocardial infarction interventricular septal rupture. A clinical case

**DOI:** 10.47487/apcyccv.v6i3.499

**Published:** 2025-09-24

**Authors:** Fernando Manuel Quevedo Candela, Gabriela Guevara Castilla

**Affiliations:** 1 Instituto Nacional Cardiovascular - INCOR, EsSalud, Lima, Peru. Instituto Nacional Cardiovascular - INCOR, EsSalud Lima Peru

**Keywords:** Heart Rupture, Post-Infarction, Ventricular Septal Rupture, Myocardial Infarction, Cardiac Surgical Procedures, Rotura cardíaca post infarto, Rotura Septal Ventricular, Infarto del Miocardio, Procedimientos Quirúrgicos Cardíacos

## Abstract

Clinical case of an 86-year-old male patient presenting with a late anterior myocardial infarction without reperfusion is reported. Initial echocardiographic assessment revealed a rare and complex mechanical complication: intramyocardial dissection of the left ventricular apex associated with interventricular septal rupture. Given the severity of the condition, an urgent surgical intervention was undertaken using a bovine pericardial patch. Although the initial postoperative course was favourable, multiple subsequent complications ultimately led to the patient’s death from ventricular arrhythmia on day 50th of hospitalisation. This report underscores the importance of timely diagnosis and multidisciplinary management of this rare clinical entity.

## RESUMEN

Se presenta el caso clínico de un paciente masculino de 86 años con infarto de miocardio anterior evolucionado y no reperfundido. La evaluación ecocardiográfica inicial reveló una complicación mecánica inusual y compleja: disección intramiocárdica del ápex ventricular izquierdo asociada a ruptura del septum interventricular. Debido a la complejidad del caso, se decidió una intervención quirúrgica urgente, empleando un parche de pericardio bovino. Aunque inicialmente la evolución fue satisfactoria, múltiples complicaciones posoperatorias condujeron finalmente al fallecimiento del paciente a causa de una arritmia ventricular a los 50 días de hospitalización. Este reporte resalta la importancia del diagnóstico oportuno y el manejo multidisciplinario de esta rara entidad clínica.

## Introduction

Myocardial infarction is a potentially life-threatening complication of coronary atherosclerosis, with in-hospital mortality rates reaching up to 10%. ^1^^-^^4^ Delays in achieving optimal reperfusion may precipitate mechanical complications that are associated with increased mortality. ^3^^,^^4^ Among these, interventricular septal rupture carries particularly high mortality rates.

This entity represents one of the major mechanical complications of myocardial infarction, with a low but clinically relevant incidence in both ST-elevation myocardial infarction (STEMI) and non-ST-elevation myocardial infarction (NSTEMI). ^5^^-^^9^ Surgical treatment is essential and is preceded by supportive medical therapy aimed at improving cardiac function. In severe cases, mechanical circulatory support devices may be required. ^10^ Nevertheless, surgical intervention carries a high risk of both short- and long-term mortality. In addition, intramyocardial dissection, an extremely rare but critical complication, may necessitate immediate surgical correction. ^11^ We report the case of an elderly patient with anterior myocardial infarction complicated by interventricular septal rupture and intramyocardial dissection, managed surgically.

## Case report

An 86-year-old man with a history of hypertension and diabetes mellitus experienced mild oppressive chest pain seven days prior to admission to our institution, which initially went unnoticed. Due to persistence of the symptom, he presented five days later to his local hospital, where the electrocardiogram showed acute ischaemic changes consistent with anterior STEMI. He was initially managed with pharmacological therapy, including dual antiplatelet loading, statins, and anticoagulation, and was subsequently referred to our institution.

**Figure 1:**
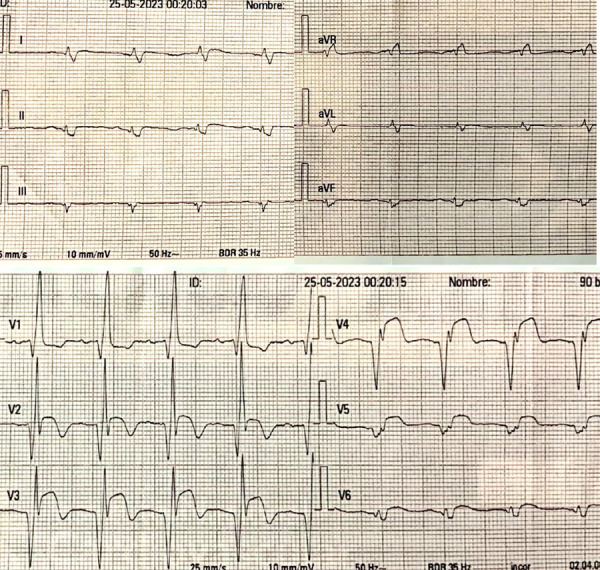
Admission electrocardiogram. Sinus rhythm, right bundle branch block pattern. ST-segment elevation and negative T waves in leads V3-V6; additionally, ST-segment depression is observed in leads II, III, and aVF.

**Figure 2:**
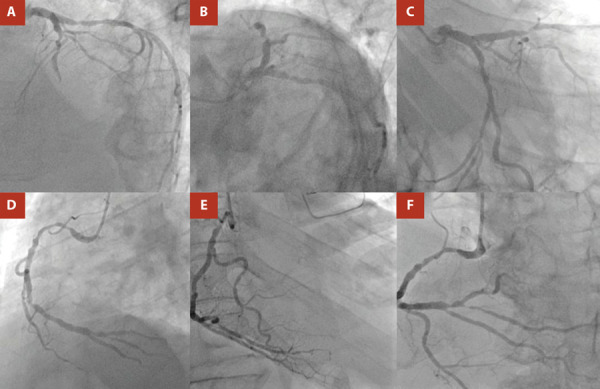
Coronary angiography. Left coronary artery (**A:** left anterior oblique cranial view; **B:** left anterior oblique caudal view [spider]; **C:** right anterior oblique caudal view). Right coronary artery (**D:** left anterior oblique cranial view; **E:** right anterior oblique cranial view; **F:** anteroposterior view). Severe multivessel coronary artery disease is demonstrated.

**Figure 3:**
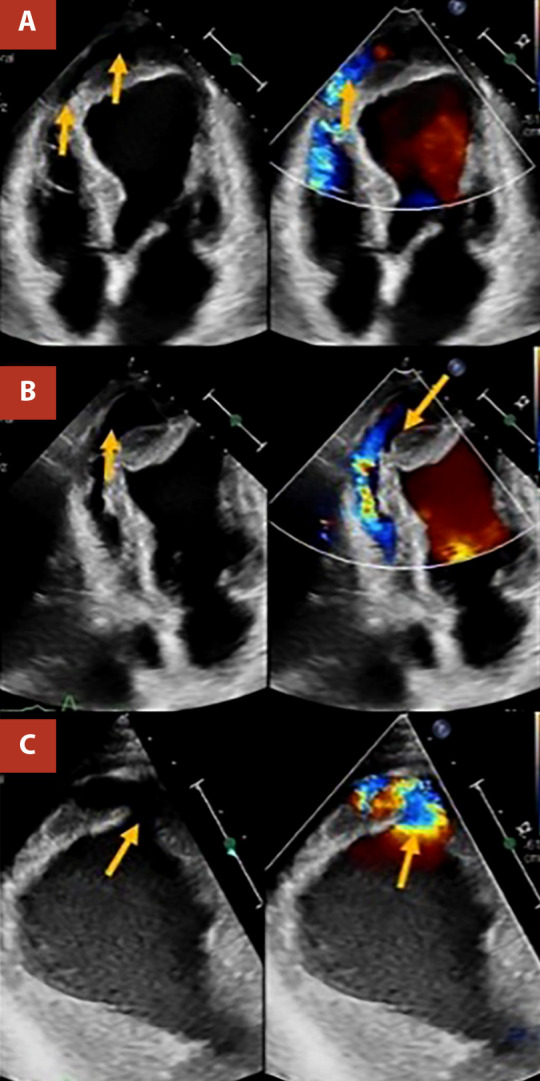
Transthoracic echocardiography on admission. Dilated left chambers with moderate concentric hypertrophy. Left ventricular ejection fraction of 34%. The apical myocardium is divided into two layers (intramyocardial dissection), demonstrated by an anechoic space with internal flow **(A)**, which communicates with the right ventricle through apical septal rupture **(B)**. Apical ventricular septal rupture measuring 3 mm **(C)**.

At admission to our institution (six days after symptom onset), the patient presented without chest pain, without the need for supplemental oxygen or vasoactive drugs, with a blood pressure of 137/78 mmHg, a heart rate of 95 beats per minute, and a respiratory rate of 18 breaths per minute. Clinical examination revealed a left parasternal, multifocal, pansystolic murmur graded IV/VI. On admission, the electrocardiogram showed extensive anterior ST-segment elevation (Figure 1), and cardiac biomarkers were elevated, with a high-sensitivity troponin T level of 0.985 ng/mL (upper reference limit: 0.053 ng/mL). Invasive coronary angiography demonstrated severe multivessel coronary artery disease (Figure 2). Transthoracic echocardiography (Figure 3) revealed akinesia in all apical segments of the left ventricle. Furthermore, the apical myocardium appeared split into two layers, separated by an anechoic space with systolic turbulent flow demonstrated by color Doppler, consistent with an intramyocardial dissection flap in the apical region. The dissection communicated with the left ventricular cavity through a 13-mm orifice located at the apex (Figure 3A, B). In addition, this intramyocardial dissection area was connected to the right ventricular apex through a 3-mm rupture in the most apical interventricular septum, resulting in a left-to-right shunt (Figure 3C).

**Figure 4:**
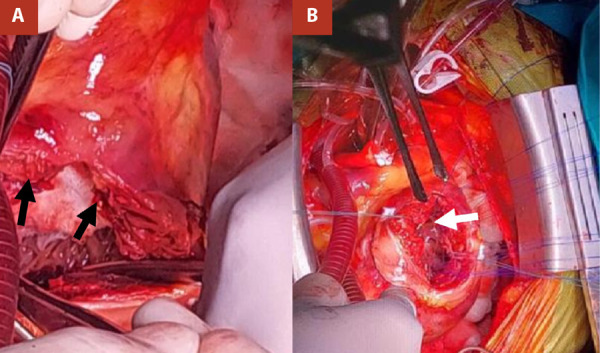
Intraoperative findings. Apical ventricular septal rupture (VSR) measuring 18 mm at the level of the dissecting left ventricular free wall (black arrows in Panel A); thrombi within the LV (White arrow in Panel B); and pericardium adherent to the VSR (black and white arrows).

**Figure 5:**
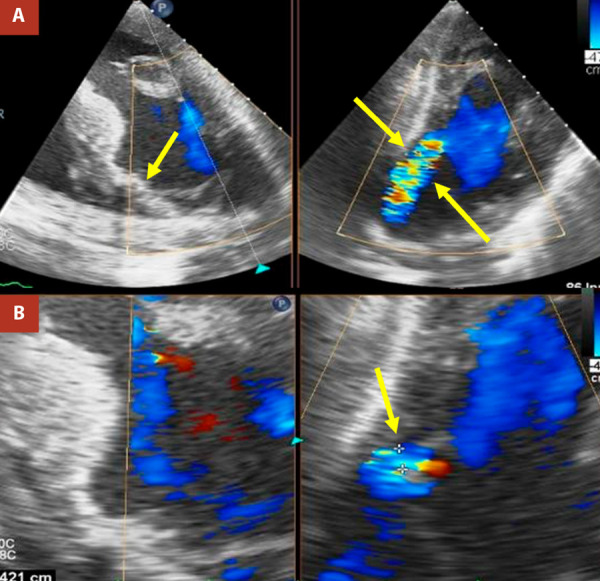
Postoperative transesophageal echocardiography. Hypertrophic left ventricle with left ventricular ejection fraction of 52%. Anterior wall akinesia. A bovine pericardial patch (BPP) is observed at the mid-apical level, excluding the apex (yellow arrow, Panel A, left). Residual shunt measuring 11 × 5 mm at the level of the BPP in the lateral wall of the left ventricle (yellow arrows in Panel A, right, and Panel B, right).

Given the complexity of the case and the high surgical risk, a multidisciplinary heart team meeting was convened, reaching consensus on the indication for emergency surgical intervention. Intraoperative transesophageal echocardiography (TEE) revealed an apical ventricular septal rupture (VSR). A myocardial dissection was identified, with a 12-mm entry orifice at the apex and a 15-mm exit orifice at the septoapical segment. Color Doppler imaging demonstrated turbulent flow throughout the entire dissected tract. These findings are documented in Figures 4 and 5.

Surgical closure of the septal defect and apical aneurysm was performed using a bovine pericardial patch (BPP), reinforced with two Teflon strips. Myocardial revascularization was not undertaken due to the prolonged cardiopulmonary bypass time (145 minutes). In the immediate postoperative period, while in the intensive care unit (ICU), the patient developed active bleeding, prompting surgical re-exploration for haemostasis control. Subsequent echocardiographic evaluation demonstrated a residual shunt measuring 11 x 5 mm in the mid-apical region of the left ventricle, corresponding to the area of the patch.

During his postoperative stay in the ICU, the patient had a complicated clinical course marked by infectious complications and issues related to prolonged mechanical ventilation. He also developed episodes of severe ventricular arrhythmias that were unresponsive to advanced cardiopulmonary resuscitation measures, ultimately resulting in a fatal outcome.

## Discussion

VSR is an uncommon mechanical complication of myocardial infarction, typically occurring between the third and fifth day after the ischaemic event. Its clinical presentation is variable, ranging from an asymptomatic left parasternal holosystolic murmur to severe manifestations such as acute heart failure or cardiogenic shock. Echocardiography is the diagnostic modality of choice, as it not only confirms the presence of VSR but also characterises its location and morphology. In anterior wall infarctions, the rupture usually occurs at the apical region and follows a single tract, whereas inferior infarctions are more frequently associated with complex defects, extensive tissue destruction, and multiple communicating tracts. ^2^^,^^3^^,^^5^^-^^10^^,^^12^

Several risk factors have been identified in association with the development of these mechanical complications, including advanced age, female sex, history of heart failure, chronic kidney disease, and first myocardial infarction, as well as the use of thrombolytic therapy, ^13^ which carries an increased risk of haemorrhagic transformation of the infarcted myocardial area.

In the case described, an 87-year-old man with a history of hypertension and diabetes mellitus was admitted with an untreated anterior myocardial infarction that progressed to VSR and intramyocardial dissection. This represents an infrequent presentation, as rupture typically extends directly into the free cavity, involving either the interventricular septum or the lateral wall of the left ventricle. In this case, however, the rupture advanced along an irregular path through the myocardial thickness, dissecting the tissue between muscle fibres. This atypical evolution highlights the complexity of post-infarction mechanical complications and underscores the importance of early detection, given the high risk of progression of dissected tissue to complete rupture.

Septal rupture is typically the result of two main mechanisms: on the one hand, progressive expansion of the infarcted area, which leads to myocardial wall weakening and increased susceptibility to rupture; and on the other, shear forces generated at the interface between the akinetic territory and the adjacent hypercontractile region, where mechanical stress is maximal. It has been reported that VSR may extend and give rise to intramyocardial dissection, or conversely, that the latter may precede and evolve into septal rupture. Both entities can progress to the formation of a pseudoaneurysm and, ultimately, cardiac rupture. In rare cases, intramyocardial dissection has been observed to resolve spontaneously, either partially or completely. ^15^^-^^17^

Reports have described the echocardiographic features of intramyocardial dissection, including the formation of a neocavity within the myocardium with a hypoechoic center, a thinned endomyocardial border surrounding the cavitary defect, preservation of myocardium outside the cystic areas, and communication between both ventricles through the dissection, which is more accurately identified with color Doppler echocardiography. ^14^^,^^18^ Similarly, transthoracic echocardiography remains the cornerstone of diagnosis, although it may be misinterpreted as a left ventricular thrombus or a ventricular pseudoaneurysm; in such cases, cardiac magnetic resonance imaging or computed tomography enables a definitive diagnosis. ^14^^,^^19^

Urgent treatment is usually required, although in selected cases a conservative strategy may be considered. Nevertheless, in-hospital mortality remains high, particularly when associated with VSR, reaching up to 78%. ^20^^,^^21^ Careful case-by-case evaluation and consideration of predictive risk factors are therefore essential to guide management decisions and improve clinical outcomes.

In conclusion, post-myocardial infarction intramyocardial dissection, when associated with mechanical complications such as VSR, markedly worsens prognosis, with high mortality rates despite surgical treatment. Early diagnosis is therefore essential, requiring thorough clinical assessment and physical examination aimed at detecting suggestive findings, together with the systematic use of imaging techniques, particularly echocardiography and cardiac magnetic resonance, to allow timely identification of these complications. In addition, recognition of risk factors associated with the development of mechanical lesions is crucial to enable close surveillance and prompt intervention. Joint evaluation by the heart team is recommended to individualize management and guide appropriate therapeutic decisions, taking into account predictors of mortality and surgical risk, with the ultimate goal of optimizing clinical outcomes.
